# Complex roles of nicotinamide *N*-methyltransferase in cancer progression

**DOI:** 10.1038/s41419-022-04713-z

**Published:** 2022-03-25

**Authors:** Weixuan Wang, Changmei Yang, Tianxiang Wang, Haiteng Deng

**Affiliations:** 1 Institute of Chinese Medicine, Guangdong Pharmaceutical University, Guangzhou, People’s Republic of China; 2 MOE Key Laboratory of Bioinformatics, Center for Synthetic and Systematic Biology, School of Life Sciences, Tsinghua University, Beijing, People’s Republic of China

**Keywords:** Cancer, Mechanisms of disease

## Abstract

Nicotinamide *N*-methyltransferase (NNMT) is an intracellular methyltransferase, catalyzing the N-methylation of nicotinamide (NAM) to form 1-methylnicotinamide (1-MNAM), in which *S*-adenosyl-l-methionine (SAM) is the methyl donor. High expression of NNMT can alter cellular NAM and SAM levels, which in turn, affects nicotinamide adenine dinucleotide (NAD^+^)-dependent redox reactions and signaling pathways, and remodels cellular epigenetic states. Studies have revealed that NNMT plays critical roles in the occurrence and development of various cancers, and analysis of NNMT expression levels in different cancers from The Cancer Genome Atlas (TCGA) dataset indicated that NNMT might be a potential biomarker and therapeutic target for tumor diagnosis and treatment. This review provides a comprehensive understanding of recent advances on NNMT functions in different tumors and deciphers the complex roles of NNMT in cancer progression.

## Facts

1. NNMT is highly expressed in various cancers while the underlying mechanisms for NNMT-mediated tumor progression remain elusive.
2. NNMT acts as a metabolic enzyme to regulate cell metabolism and can trigger epigenetic remodeling in kidney and ovarian cancers.
3. NNMT regulates tumor progression in a context-dependent manner.

## Open questions

1. The relationship between the expression of NNMT and NAM metabolism in different organs and tissues under normal physiological conditions remains to be clarified.
2. NNMT-mediated NAM-metabolome including SAM, SAH, NAD^+^, and acetyl-CoA and its effects on epigenome need to be characterized in different cancer tissues.
3. It is important to examine the effects of 1-MNAM on tumor cells and tumor microenvironment.
4. The development and validation of NNMT inhibitors in tumor therapy is needed.

## Introduction

**Fig. 1:**
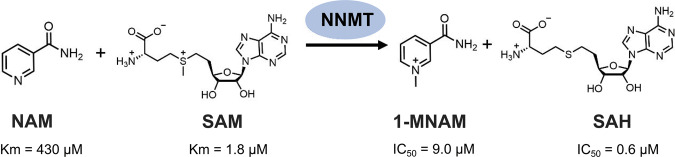
NAM N-methylation reaction catalyzed by NNMT. NNMT catalyzes the methylation of NAM to 1-MNAM by using SAM as a co-substrate. Human liver NNMT has apparent Km values for NAM and SAM, of 430 and 1.8 μM, respectively. 1-MNAM and SAH inhibit the methyltransferase activity of NNMT with IC_50_ values of 9.0 and 0.6 μM, respectively.

Nicotinamide *N*-methyltransferase (NNMT) is an *S*-adenosyl-l-methionine (SAM)-dependent cytosolic enzyme. Taking SAM as the methyl donor, NNMT catalyzes N-methylation of nicotinamide (NAM) to generate 1-methylnicotinamide (1-MNAM) and *S*-adenosyl-l-homocysteine (SAH) (Fig. 1). Evidence accumulates that NNMT mRNA and protein levels are elevated in various human cancers, and enhanced NNMT expression has been associated with tumor progression [1]. As a metabolic enzyme, NNMT is closely linked to the levels of NAM, SAM, 1-MNAM, and SAH in tumor cells. However, it is unclear how NNMT affects cell metabolism and signaling pathways in different tumors. How NNMT promotes tumor progression and suppression is poorly elucidated as well. Besides, 1-MNAM has been considered as an inactive metabolite for a long time, which turned out to be inaccurate. Actually, it is uncontestable that NNMT plays a complex role in different tumors due to their diverse backgrounds and contexts.

In this review, we intend to provide a comprehensive overview of the roles and functions of NNMT in different tumor types. We summarized recent advances of NNMT and analyzed its potential effects on cell metabolism or signaling pathways in disparate tumors case-by-case. In addition, we proposed several possible mechanisms of NNMT which are involved in tumor development. Our analysis indicates that NNMT is of great potency to be developed as a therapeutic target in some cancers such as kidney renal clear cell carcinoma, pancreatic adenocarcinoma, ovarian cancers, and further studies are needed to fully assess the potential clinical value of NNMT.

## Cloning, characterization, and tissue distribution of NNMT

The substrate of NNMT, NAM is an amide derivative of nicotinic acid (NA), also known as vitamin B3, and is mainly used to treat pellagra, stomatitis as well as glossitis. Interest in NAM methylation was prompted in the 1940s when methylated vitamin B3 metabolites were discovered in the urine [2]. In 1951, Giulio Cantoni purified NNMT partially and characterized its methyltransferase activity for the first time [3]. The next year, SAM was identified as the methyl donor for NAM methylation, and it was also characterized as a universal methyl donor participating in most methylation reactions in cells [4]. Four decades later, Weinshilboum et al. cloned and expressed the *NNMT* gene in human liver tissue in vitro [5]. *NNMT* gene is located on human chromosome 11, containing three exons and two introns, with a total length of 16.5 kb. The transcriptional mRNA contains 1578 bases, and the encoded protein has 264 amino acids with a molecular weight of 29.6 kDa. Alternative splice variants of NNMT were also unveiled by RNA sequencing, but they account for a negligible proportion and their functions are poorly defined (www.gtexportal.org/). In addition to NAM, NNMT also methylates structural analogs of NAM, such as pyridines, quinoline, isoquinoline, thionicotinamide, and others [6].

**Fig. 2:**
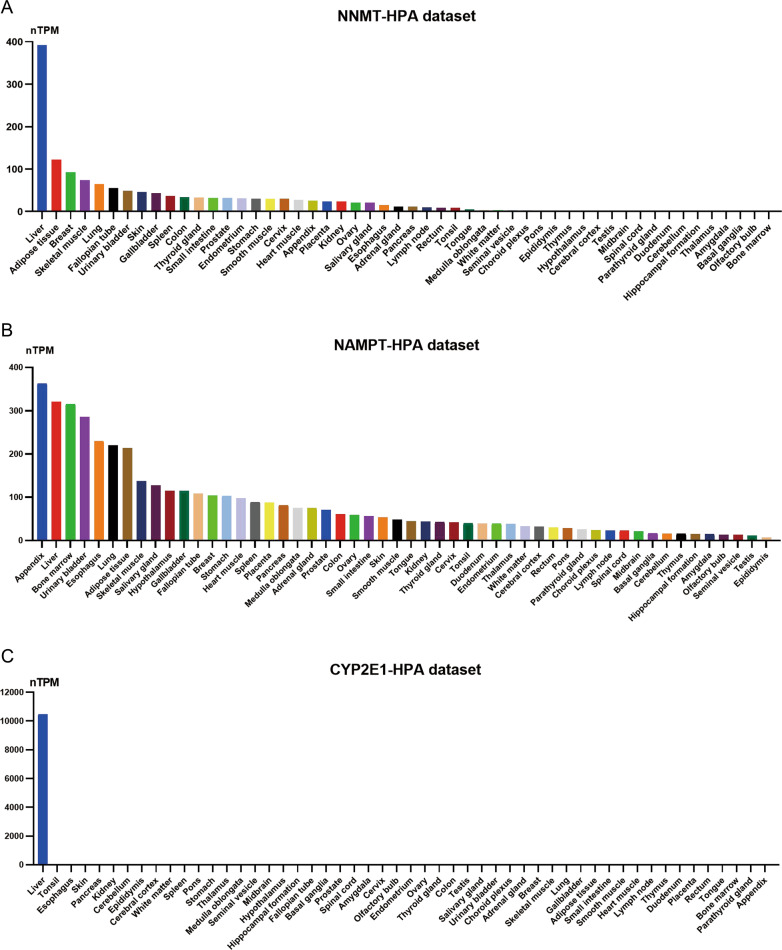
RNA expression levels of NNMT, NAMPT, and CYP2E1 in different human tissues. Human *NNMT* and *CYP2E1* are preferentially expressed in the liver while *NAMPT* has low tissue specificity. **A** RNA expression of *NNMT* in different tissues from the Human Protein Atlas dataset (https://www.proteinatlas.org/). The expression of *NNMT* is the highest in the liver, and it is also expressed in cardiocytes, smooth muscle cells, adipocytes, cervix, lung, brain, colon, bladder, testis, and placenta to a lesser extent. **B** RNA expression of *NAMPT* in different tissues from the Human Protein Atlas dataset. *NAMPT* is expressed in the appendix, liver, and other tissues, which has low tissue specificity. **C** RNA expression of *CYP2E1* in different tissues from the Human Protein Atlas dataset. *CYP2E1* is highly expressed in the liver and it is barely expressed in other tissues. nTPM normalized protein-coding transcripts per million.

NNMT is predominantly expressed in the liver, and it is also expressed in cardiocytes, smooth muscle cells, adipocytes, cervix, lung, brain, colon, bladder, testis, and placenta to a lesser extent [7] (Fig. 2A). The highest expression level of NNMT in the liver indicates that NNMT is important in maintaining NAM homeostasis in hepatocytes. Nicotinamide phosphoribosyltransferase (NAMPT, Fig. 2B), NNMT, and Cytochrome P450 2E1 (CYP2E1, Fig. 2C) are three known NAM-consuming enzymes. Based on their Km values, we can deduce that NAM is supplied for NAMPT (Km = 1 μM)-catalyzed NAD^+^ synthesis when it is at a relatively low level in the liver. When the NAM level increases, the enzymatic activity of NNMT (Km = 430 μM) is upregulated to clear excess NAM and remove the inhibition effect of NAM on PARPs, sirtuins, or others. More extremely, when NAM is at an acute pharmacological dose, it is mainly converted to NAM N-oxide by CYP2E1 (Km = 2.98 mM) as quickly as possible [8–10]. In adipose tissue, perhaps the most important thing for adipocytes is not producing energy through mitochondria but storing fat by consuming NAM which is vital for mitochondria biogenesis and NAD^+^ synthesis. Meanwhile, adipocytes retard polyamine flux to accumulate acetyl-CoA for lipid synthesis by consuming SAM with a high level of NNMT, which is considered as a major methyltransferase in adipocytes [11]. Consistent with this, a high level of NNMT expression in human adipose tissue positively correlates with adiposity and insulin resistance [12, 13]. Intriguingly, NNMT is also highly expressed in cardiocytes and smooth muscle cells where mitochondria functions and NAD^+^ are indispensable. The possible mechanism is that NAD^+^ synthesis and breakdown are kept at a relative equilibrium state by NNMT to avoid superfluous NAD^+^ synthesis from NAM in these cells. Another possible reason is that NNMT may deplete redundant NAM in cardiocytes and smooth muscle cells, to enhance PARPs activities for DNA repair. On the whole, the concrete roles played by NNMT in different cell types remain to be dissected on a case-by-case basis.

## Structure, enzyme activity, and regulation of NNMT

NNMT protein is highly conserved across mammals, with 85% amino acid homology between humans and mice [14]. Weinshilboum et al. found that human liver NNMT had Km values of 430 and 1.8 μM for NAM and SAM respectively by using the radiochemical enzymatic assay at 37 °C, which is based on the conversion of NAM to radioactively labeled 1-MNAM with [methyl-^14^C]-SAM as the methyl donor [5]. In 2016, Martin et al. determined the kinetic parameters of NNMT-catalyzed NAM methylation reaction by using a UHP-HILIC-MS (ultra-high-performance hydrophilic interaction liquid chromatography–mass spectrometry) method, in which Km values for NAM and SAM were determined as 200 and 8.5 μM, respectively [15]. A recent study reported that the Km values of NNMT for NAM and SAM was 20 ± 3 μM and 24 ± 6.8 μM via SAH hydrolase (SAHH)-coupled fluorescent assay [16], which was quite different from those documented in previous literature. The discrepancies were possibly attributable to different experimental conditions such as temperature, pH, additives, and ionic strength. Measurements with the UHP-HILIC-MS method employed 1 mM dithiothreitol that can disrupt the disulfide bonds in NNMT protein and change the three-dimensional structure, while the SAHH-coupled fluorescent assay employed a complex reaction buffer containing SAHH, adenosine deaminase, ThioGlo, MgCl_2_, potassium phosphate, EDTA that potentially interfere the reaction kinetics [17].

The crystal structure of the “hNNMT-NAM-SAH” ternary complex at 2.7 Å was first determined by Emanuelli using X-ray crystal diffraction. They found the binding sites of NAM and SAH in NNMT were adjacent to each other and D197 and Y20 in NNMT protein are indispensable for NAM methylation [18]. To the best of our knowledge, the crystal structure of the “hNNMT-NAM-SAM” ternary complex or “hNNMT-NAM-SAM-SAH” quaternary complex has not been resolved yet. However, Jian Jin et al. solved the crystal structure of hNNMT in combination with bisubstrate inhibitor MS2734 which has a linker that covalently connected NAM and SAM mimic moieties [16]. MS2734 inhibited the methyltransferase activity of NNMT with IC_50_ 14 ± 1.5 μM. An increasing number of NNMT inhibitors have been developed in recent years as Table 1 displays [19–31]. In addition, protein posttranslational modifications also regulate the enzymatic activity of NNMT. For instance, citrullination of R132 by protein arginine deiminase 1 or 2 (PAD1 or PAD2) inactivates NNMT, while phosphorylation of Y11 and S108 can activate NNMT [32, 33]. A quantitative analysis of succinylation in HeLa cells showed that NNMT has two succinylation sites on K8 and K23, but the functions of succinylation on NNMT have not been well investigated [34]. We summarized the known posttranslational modifications and sites on NNMT in Table 2, which were obtained from the iPTMnet Website (https://research.bioinformatics.udel.edu/iptmnet/).

**Table 1:** Inhibitors of NNMT.

Type of inhibitors	Name	K_i_ (nM)	IC_50_ (μM)	Ref
Metal ion	Cu^2+^/Cd^2+^/Mn^2+^	–	–	[19]
Products	1-MNAM	–	9.0 ± 0.6	[20]
	SAH	–	0.6	[21]
Product analogs	BrSAH	750 ± 50	1.5	[21]
	6-methylamino-nicotinamide	–	19.81 ± 2.50	[22]
Broad-spectrum MTase inhibitor	Sinefungin	–	3.9 ± 0.3	[15]
Covalent inhibitor	RS004	–	1.0	[23]
NAM analogs	JBSNF-000265	–	0.588 ± 0.075	[24]
	JBSNF-000088	–	1.8	[25]
N-methylated quinoliniums (substrate mimetic inhibitors)	1-MQ	–	12.1 ± 3.1	[20]
	5-amino-1-MQ	–	1.2	[20]
	8-methyl-1-MQ		1.8 ± 0.5	[20]
	CH9NH2	–	1.2	[20]
Amino-bisubstrate inhibitors	MS2734	–	14 ± 1.5	[26]
	MS2756	–	160 ± 1	[16]
	Compound 45	–	29	[27]
	Compound 78	–	1.41	[22]
	NS1	0.5	–	[26]
	LL320	1.6 ± 0.1	–	[28]
Others	Pyrimidine-5-carboxamide compounds	–	0.057–0.385	[29]
	Cyclic peptide	–	0.229 ± 0.007	[30]
	AK-4/AK-12/AK-23	–	0.008, 0.07, 0.15	[31]

**Table 2:** Posttranslational modifications of NNMT.

PTM Types	Sites
Ubiquitination	K8, K23, K26, K39, K43, K47, K96, K100, K123, K136, K210
Acetylation	M1, K8, K39, K43
Phosphorylation	S3, Y11, Y24, Y25, S35, S108, Y113, Y203, Y204
Methylation	K8
Citrullination	R132
Succinylation	K8, K23

## The interactome of NNMT

We examined the interacting proteins of NNMT using the STRING database [35] (https://cn.string-db.org/), showing that NNMT interacts with the following proteins including caveolae-associated protein 1 (PTRF), aldehyde oxidase (AOX1), epidermal growth factor receptor (EGFR), four and a half LIM domains protein 2 (FHL2), nicotinamide phosphoribosyltransferase (NAMPT), NAD-dependent protein deacetylase sirtuin 1-7 (SIRT1-7), ADP-ribosyl cyclase/cyclic ADP-ribose hydrolase 2 (BST1), and ADP-ribosyl cyclase/cyclic ADP-ribose hydrolase 1 (CD38) in human and mice. AOX1 oxidizes 1-MNAM into two related compounds, *N*1-methyl-2-pyridone-5-carboxamide (2py) and *N*1-methyl-4-pyridone-3-carboxamide (4py), and all three metabolites are excreted in the urine [11]. Therefore, we propose that the interaction of NNMT and AOX1 facilitates the clearance of excessive NAM. Intriguingly, the interaction of NNMT and NAMPT implies that NAM elimination and NAD^+^ synthesis from NAM occur concurrently. That suggests NNMT and NAMPT have synergy effects on the regulation of NAD^+^ levels in cells. All sirtuins are NNMT binding partners while their roles in tumor progression remain to be characterized. Moreover, the interactions between NNMT with CD38 or BST1 that are NAD^+^-degrading enzymes suggest that they work coordinately to regulate cellular NAD^+^ levels. CD38 and BST1 also synthesize the second messengers cyclic ADP-ribose, proposing that NNMT plays a role in signal transduction by binding with CD38 or BST1.

In addition to the STRING database, we summarized NNMT binding proteins by searching literature from PubMed (https://pubmed.ncbi.nlm.nih.gov/). It was reported that RAN-binding protein 9 (RANBP9) interacted with NNMT by yeast two-hybrid screening [36]. RANBP9 is an adapter protein that interacts with proto-oncogene hepatocyte growth factor receptor (MET) to activate the Ras and PI3K/AKT signaling pathways [37, 38]. Their interaction indicated that NNMT was associated with PI3K/AKT pathway. Co-IP and confocal immunofluorescence experiments showed that NNMT interacted and co-localized with alpha-tocopherol transfer protein-like (TTPAL), which was upregulated and correlated with poor survival in gastric cancer [39]. Overexpression of TTPAL in gastric cancer cells upregulated NNMT, indicating that TTPAL stabilized NNMT in the PI3K/AKT signaling pathway [39]. In addition, Co-IP followed by mass spectrometry of mouse liver showed that the NNMT-interacted with betaine-homocysteine methyltransferase (BHMT), fructose 1,6 bisphosphatase 1 (FBP1), dihydroxyacetone kinase (DAK), methionine adenosyltransferase 1a (MAT1A), heat shock protein A8 (HSPA8), solute carrier transporter mitochondrial (citrate) (SLC25A1), selenium binding protein 2 (SELENBP2), and adenosylhomocysteinase (AHCY) [40]. The interactions between NNMT and three methionine cycle-related proteins AHCY, BHMT, and MAT1A were validated through Co-IP followed by immunoblotting in mouse liver and 293 T cells [40]. The proximity of these proteins may enhance the recycling of homocysteine back to SAM. More importantly, these interactions may be critical in the NNMT-regulated epigenome. Moreover, PAD1 and PAD2 that catalyze citrullination of R132 of NNMT [32], play a role to regulate the methyltransferase activity of NNMT. The NNMT interactome is summarized in Table 3.

**Table 3:** The interacting proteins of NNMT.

Name	Methods for identification of protein interactions	Species
PTRF	Co-expression	*Homo sapiens*
AOX1	Co-expression, Database, Textmining	*Homo sapiens; Mus musculus*
EGFR	Co-expression, Textmining	*Homo sapiens*
FHL2	Co-expression	*Homo sapiens*
NAMPT	Database, Textmining	*Homo sapiens; Mus musculus*
SIRT1	Database, Textmining	*Homo sapiens*
SIRT5	Co-expression, Database, Textmining	*Homo sapiens; Mus musculus*
SIRT4	Database, Textmining	*Homo sapiens; Mus musculus*
SIRT7	Database, Textmining	*Homo sapiens; Mus musculus*
SIRT3	Database, Textmining	*Homo sapiens; Mus musculus*
BST1	Database, Textmining	*Mus musculus*
CD38	Database, Textmining	*Mus musculus*
SIRT2	Database, Textmining	*Mus musculus*
SIRT6	Database, Textmining	*Mus musculus*
RANBP9	Yeast two-hybrid screening	*Homo sapiens*
TTPAL	Co-IP	*Homo sapiens*
BHMT	Co-IP	*Homo sapiens; Mus musculus*
FBP1	Co-IP	*Mus musculus*
DAK	Co-IP	*Mus musculus*
MAT1A	Co-IP	*Mus musculus*
HSPA8	Co-IP	*Mus musculus*
SLC25A1	Co-IP	*Mus musculus*
SELENBP2	Co-IP	*Mus musculus*
AHCY	Co-IP	*Mus musculus*
PAD1	AP-MS; Enzyme activity measurement	*Homo sapiens*
PAD2	AP-MS; Enzyme activity measurement	*Homo sapiens*

*Co-IP* co-immunoprecipitation, *AP-MS* affinity purification-mass spectrometry.

## NNMT-associated metabolome and epigenetics

**Fig. 3:**
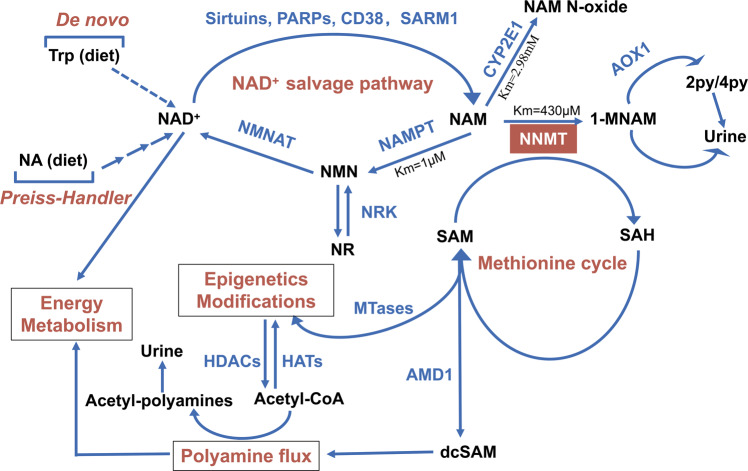
Metabolic processes mediated by NNMT. NAM is N-methylated by NNMT consuming the universal methyl donor SAM. The methylated product 1-MNAM is further oxidized by AOX1 into two related compounds 2py and 4py. All three metabolites are eventually excreted in the urine but the major urinary metabolites are 1-MNAM and 2py, accounting for 20–35% and 45–60%, respectively. When NAM is at an acute pharmacological dose, it is mainly converted to NAM N-oxide by CYP2E1. NAM is a crucial precursor for NAD^+^, which is one of the most abundant molecules in the human body, needed in ~500 different enzymatic reactions and present at about three grams in the average person. It can be synthesized from several distinct dietary precursors through three separate pathways, de novo, Preiss–Handler, and salvage pathways. SAM donates methyl groups for epigenetics modifications as well as provides propylamine groups in polyamine flux. AMD1 uses SAM to generate dcSAM for synthesizing spermidine and spermine. Then, SSAT takes acetyl-CoA as a substrate to acetylate spermidine and spermine and form acetylpolyamine. SAM and acetyl-CoA are substrates for histone methylation and acetylation and as a result, levels of SAM or acetyl-CoA may affect epigenetics modifications. Trp tryptophan, NAM nicotinamide, NA nicotinic acid, dcSAM decarboxylated SAM, 2py *N*1-methyl-2-pyridone-5-carboxamide, 4py *N*1-methyl-4-pyridone-3-carboxamide, NAMPT nicotinamide phosphoribosyltransferase, NMNAT nicotinamide mononucleotide adenylyl transferases, NRK nicotinamide riboside kinase. PARPs poly (ADP-ribose) polymerases, MTases methyltransferases, HATs histone acetyltransferase, HDACs histone deacetylases, AMD1 adenosylmethionine decarboxylase, AOX1 aldehyde oxidase 1, SSAT spermidine-spermine *N*1-acetyltransferase, PARPs poly [ADP-ribose] polymerases, SARM1 sterile alpha and Toll/interleukin-1 receptor motif-containing protein 1.

As shown in Fig. 3, NNMT catalyzes the N-methylation of NAM by consuming SAM, thus regulating metabolic processes associated with NAM and SAM. NNMT is involved in NAD^+^-related signaling pathways, folate and methionine cycles, polyamine flux, and chromatin remodeling. NAD^+^ is one of the most abundant molecules in the human body, participating in hundreds of redox reactions in metabolic pathways, including glycolysis, TCA cycle, oxidative phosphorylation, and other basic biological energy pathways [41]. It is uncontestable that NAD^+^ plays multifaceted roles in signaling pathways involved in DNA repair, cell division, chromatin remodeling, and epigenetics under stress conditions [42]. Specifically, NAM, by itself or through altering NAD^+^ levels, modulates mitochondrial functions and activities to affect cell viability and metabolism [43]. NNMT knockdown in mouse adipocytes significantly increased intracellular NAD^+^ levels, silencing NNMT in HT-29 cells led to a 30% rise of NAD^+^ levels approximately, whereas NNMT overexpression in SW480 cells led to a 30% decrease in intracellular NAD^+^ levels [44, 45]. Our recent research showed that NAD^+^ decline promoted epithelial-mesenchymal transition (EMT), migration, and invasion of cancer cells by activating the STAT3 signaling pathway and oxidatively degrading 15-hydroxyprostaglandin dehydrogenase (15-PGDH, an NAD^+^-dependent enzyme) via accumulated ROS. 15-PGDH degradation leads to PGE2 accumulation and excretion to the tumor microenvironment, resulting in aggravating inflammation [46, 47]. Consistently, the replenishment of NAD^+^ precursors, NA or NAM, can reverse this phenotype. It remains to be elucidated whether NNMT can promote EMT and PGE2 production in cancer cells by depleting NAD^+^.

In mesenchymal cancer stem cells, NNMT overexpression depleted intracellular NAM and therefore enhanced the activity of PARP1, increasing the chemoradiotherapy resistance of cancer cells [48]. Additionally, overexpression of NNMT increased the levels of sirtuin 1 (SIRT1) in prostate and breast cancer cells, eventually promoting cell migration, invasion, and enhancing chemoresistance of cancer cells [49, 50]. Hong et al. reported that 1-MNAM can stabilize SIRT1 by decreasing its ubiquitination [51]. In aggregate, it is likely that NNMT enhances SIRT1 activity by decreasing NAM and increasing 1-MNAM, and enhances PARP1 activity by consuming NAM. Nevertheless, the underlying mechanisms by which NNMT regulates SIRT1 require further investigation and so does whether NNMT alters the activity of other sirtuins. When it comes to 1-MNAM, an often-overlooked product of vitamin B3 elimination, a newly published study found that 1-MNAM secreted by NNMT-expressing tumor cells was elevated in T cells and induced T cells to secrete the tumor-promoting cytokine tumor necrosis factor α (TNFα) in human ovarian cancer [52]. Elevation of NNMT in tumor cells accumulates abundant 1-MNAM while the downstream target proteins of 1-MNAM are still sealed. Apparently, more studies are needed to explore the effects and mechanisms of 1-MNAM on cancers.

SAM, another substrate of NNMT, not only provides methyl groups for histone and DNA methylation but also provides propylamine groups for polyamine biosynthesis. Polyamines are organic polycations that play a pivotal role in cell growth, aging, and cancer [53]. In polyamine metabolism (Fig. 3), SAM is utilized by adenosylmethionine decarboxylase 1 (AMD1) to generate decarboxylated SAM (dcSAM) for the synthesis of spermidine and spermine. Spermidine-spermine *N*^1^-acetyltransferase (SSAT) takes acetyl-CoA as a substrate to acetylate spermidine and spermine. Prior literature has shown that NNMT silencing in mouse adipocytes significantly upregulated intracellular SAM levels and promoted the expression and activity of ornithine decarboxylase (ODC) and SSAT by increasing the methylation level of histone H3K4, thereby promoting polyamine metabolism and energy consumption [44]. The activation of polyamine flux in adipocytes facilitated the polyamine acetylation reaction by using acetyl-CoA, ultimately leading to the loss of fat [44]. Acetyl-CoA is not only a precursor for fatty acids and cholesterol synthesis but also functions as a substrate in histone acetylation. Neither the relationship of NNMT and the ratio of acetyl-CoA/CoA, nor the links between NNMT and histone acetylation in tumor cells have been reported yet. However, NNMT does affect the ratio of SAM/SAH and histone methylation. It was documented that NNMT overexpression in 769-P cells resulted in a decrease in overall histone H3 methylation while silencing NNMT in SKOV3 cells caused an increase in overall histone H3 methylation [54]. Based on this discovery, Cravatt et al. proposed that NNMT actually acted as a regulator for the methyl donor sink in cells and its overexpression contributed to a ~40–50% decrease in H3K4me3, H3k9me2, and H3K27me3 levels, but it contributed little to arginine or DNA methylation [54]. Of note, lysine methyltransferases (KMTs) have higher Km values for SAM than protein arginine methyltransferases (PRMTs), hence the overall lysine methylation levels on H3 are easier to be reduced when SAM/SAH ratio fluctuates under the condition of cofactor competition by NNMT [55]. Interestingly, It is unexpectedly that DNA methylation was unaltered by NNMT overexpression based on published Km and IC_50_ values of human DNMT1 for SAM and SAH [56]. Even so, it is necessary to figure out the activities of which methyltransferases are changed under the fluctuation of SAM/SAH. More studies are needed to fully explore which genes or proteins are affected by these methyltransferases.

## NNMT expression in different tumors

**Fig. 4:**
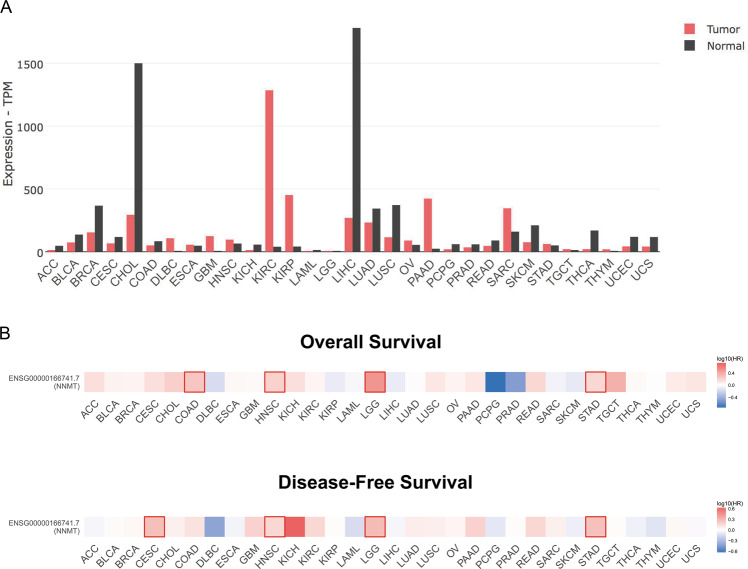
NNMT expression in different tumor types. Data is processed by GEPIA2 (http://gepia2.cancer-pku.cn/#index), a web server for cancer and normal gene expression profiling and interactive analyses. **A** The gene expression profile across all 31 types of tumor samples and paired normal tissues. The height of the bar represents the median expression of a certain tumor type or normal tissue. TPM represents transcripts per million. **B** Survival analysis based on the expression status of NNMT in 31 types of tumors. HR hazard rate, ACC adrenocortical carcinoma, BLCA bladder urothelial carcinoma, BRCA breast invasive carcinoma, CESA cervical squamous cell carcinoma and endocervical adenocarcinoma, CHOL cholangiocarcinoma, COAD colon adenocarcinoma, DLBC lymphoid neoplasm diffuse large B-cell lymphoma, ESCA esophageal carcinoma, GBM glioblastoma multiforme, HNSC head and neck squamous cell carcinoma, KICH kidney chromophobe, KIRC kidney renal clear cell carcinoma, KIRP kidney renal papillary cell carcinoma, LAML acute myeloid leukemia, LGG brain lower grade glioma, LIHC liver hepatocellular carcinoma, LUAD lung adenocarcinoma, LUSC lung squamous cell carcinoma, MESO mesothelioma, OV ovarian serous cystadenocarcinoma, PAAD pancreatic adenocarcinoma, PCPG pheochromocytoma and paraganglioma, PRAD prostate adenocarcinoma, READ rectum adenocarcinoma, SARC sarcoma, SKCM skin cutaneous melanoma, STAD stomach adenocarcinoma, TGCT testicular germ cell tumors, THCA thyroid carcinoma, THYM thymoma, UCEC uterine corpus endometrial carcinoma, UCS uterine carcinosarcoma, UVM uveal melanoma.

**Fig. 5:**
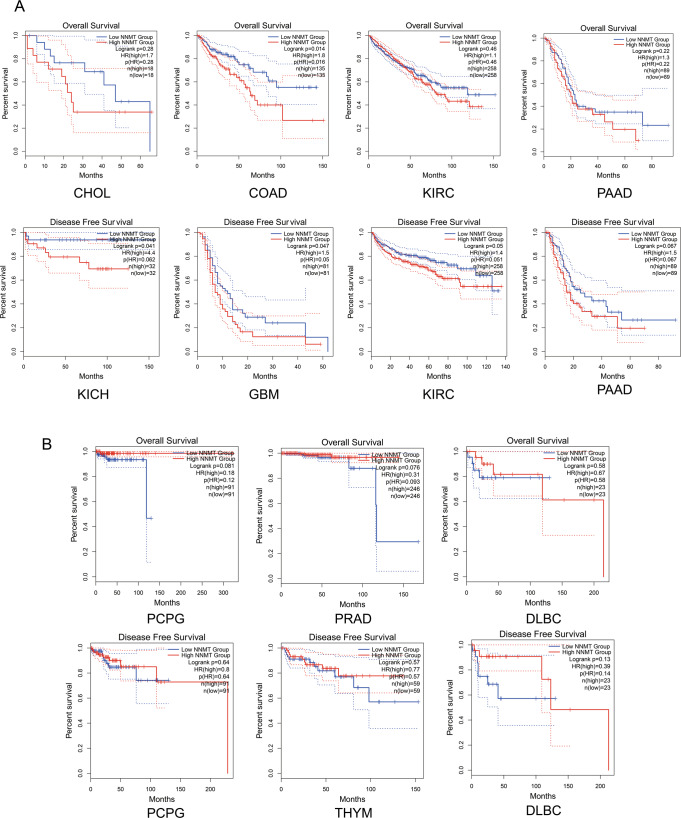
Correlation analysis of NNMT expression with OS and DFS in different cancer patients. Data were processed by GEPIA2 (http://gepia2.cancer-pku.cn/#index), a web server for cancer and normal gene expression profiling and interactive analyses. **A**, **B** Kaplan–Meier survival plots for certain tumor types.

Compared to the paired normal tissues, *NNMT* expression in 31 types of tumor samples was analyzed using datasets from The Cancer Genome Atlas (TCGA) and Genotype-Tissue Expression (GTEx). NNMT was found to be upregulated in kidney renal clear cell carcinoma (KIRC), kidney renal papillary cell carcinoma (KIRP), pancreatic adenocarcinoma (PAAD), glioblastoma multiforme (GBM), sarcoma (SARC), and lymphoid neoplasm diffuse large B-cell lymphoma (DLBC) [57]. High NNMT expression at mRNA or protein level was also reported in oral squamous cell carcinoma (OSCC) [58], gastric cancer [59], ovarian cancer [60], and even in cancer-associated fibroblasts (CAFs) of high-grade serous carcinoma (HGSC) [61], suggesting that high NNMT expression is needed for tumor progression in above-mentioned tumors. In contrast, the low NNMT expression was reported in liver hepatocellular carcinoma (LIHC), adrenocortical carcinoma (ACC), cholangiocarcinoma (CHOL), kidney chromophobe (KICH), pheochromocytoma and paraganglioma (PCPG), thyroid carcinoma (THCA), and skin cutaneous melanoma (SKCM) [57] (Fig. 4A). Accordingly, the correlation analysis for NNMT expression in 31 tumors with overall survival (OS) and disease-free survival (DFS) is shown in Fig. 4B. Apparently, the Kaplan–Meier survival analysis illustrated that OS of NNMT^high^ patients with COAD, KIRC, CHOL, PAAD, etc. was notably shorter than that of NNMT^low^ patients, and DFS of NNMT^high^ patients with KICH, KIRC, GBM, PAAD, etc. was apparently shorter than that of NNMT^low^ patients (Fig. 5A). Inversely, OS for NNMT^high^ patients with PCPG, PRAD, and DLBC was longer than that of NNMT^low^ patients, and DFS of NNMT^high^ patients with PCPG, THYM, DLBC, etc. was apparently longer than that of NNMT^low^ patients [57] (Fig. 5B). These data indicated that NNMT plays complex roles in cancer progression and it functions differently in different cancers.

### High NNMT expression promotes tumor progression in KIRC

NNMT is highly expressed in kidney renal clear cell carcinoma (KIRC) [62]. Previous reports showed that NNMT was one of the most hypomethylated genes in KIRC [63, 64]. High expression of NNMT-promoted migration and invasion of KIRC while NNMT knockdown inhibited the growth and metastasis of KIRC cells [36, 65]. The possible mechanism of NNMT-promoted invasion may be through PI3K/Akt/SP1/MMP-2 pathway in KIRC [36]. KIRC is aggressive cancer and many studies have uncovered changes in metabolic pathways that control epigenetics and biosynthesis in KIRC. For instance, aerobic glycolysis, carnitine and lipid synthesis, tryptophan metabolism are upregulated, whereas the urea cycle and tricarboxylic acid (TCA) cycle are downregulated [66]. Besides, a proteomics study showed decreased levels of enzymes involved in fatty acid β-oxidation of KIRC tissues [67]. These changes are advantageous for KIRC cells to provide building blocks that are required for cell proliferation in the absence of nutrients and oxygen. In addition, our previous studies found that HSP60, the major ATP-dependent chaperone in mitochondria that plays a crucial role in the maintenance of mitochondrial proteostasis, was downregulated in KIRC tissues. Downregulation of HSP60 disrupted mitochondrial proteostasis and enhanced tumor progression in KIRC cells [68]. The decreased mitochondrial respiratory capacity in KIRC rendered cells more sensitive to glycolytic inhibition [69]. Furthermore, HSP60 silencing induced glutamine addiction in KIRC cells for supporting nucleotide synthesis and eliminating ROS generation upon mitochondrial dysfunction, which promoted cell proliferation in KIRC [70].

To put it simply, KIRC is characterized by a typical Warburg-like phenotype, mitochondrial dysfunction, and elevated fat deposition [71]. We surmise that, on the one hand, KIRC cells may obtain more acetyl-CoA for lipid synthesis because NNMT upregulation can restrain polyamine catabolism by downregulating SAM levels, where MAT1A (S-adenosylmethionine synthase isoform type-1) and MAT2A (*S*-adenosylmethionine synthase isoform type-2) expression levels are similar to normal kidney cells [57]. However, the levels of acetyl-CoA and polyamines in KIRC cells are currently unknown. On the other hand, mitochondrial functions are lost in KIRC cells and they do not require too much NAD^+^ synthesized from NAM. This assumption can be partially supported by the evidence that the expression of rate-limiting enzyme for synthesizing NAD^+^, NAMPT, is not altered in KIRC patients while expression of NAPRT (Nicotinate phosphoribosyltransferase) and QPRT (Quinolinate phosphoribosyltransferase) is downregulated. It is worth noting that NAM can promote mitochondria biogenesis, which is unfavorable for KIRC cells maintaining their features [43]. Given that, NNMT overexpression is not bad for KIRC cells on NAD^+^ synthesis through salvage pathway but it’s probably good for fat storage and maintaining those properties of KIRC cells by consuming NAM. In line, patients with higher NNMT had shorter OS and DFS time (Fig. 5A), suggesting that NNMT is a potential indicator for poor prognosis in KIRC.

### NNMT is preferentially overexpressed in glioblastoma stem cells

Glioblastoma multiforme is a highly aggressive glial cancer with a median survival time of only 18 months [72]. It is composed of heterogeneous cell types including a subset of glioblastoma stem cells (GSCs), which are thought to sustain tumor growth. NNMT is overexpressed in GBM, preferentially in GSCs [73]. With the approach of deep learning to discover genes with prognostic value for GBM patients’ survival, researchers found that NNMT was one of the top ten ranked genes with the highest impact on chemoradiotherapy resistance in GSCs [74]. NNMT-expressing GSCs contain lower levels of methionine, SAM, NAM, and higher levels of SAH, 1-MNAM, as well as NAD^+^ unexpectedly. It has been proven that NNMT has a dual mechanism to sustain a DNA hypomethylation state through a high SAH/SAM flux and downregulation of DNMT1 and DNMT3A in a methionine-dependent manner [73]. Concerning NAD^+^ upregulation in GSCs, we can surmise that in this case, GSCs need higher levels of NAD^+^ because they are cells under replicative stress that have high demands on NAD^+^ supply for repairing DNA, maintaining self-renewal capacity, and inducing tumor plasticity [75]. This conjecture is supported by the fact that NAMPT is upregulated in GSCs, implying an active salvage pathway to produce NAD^+^. Overall, GSCs may need lower SAM levels to maintain DNA or histone hypomethylation state for their undifferentiated features. Meanwhile, they rely on large amounts of NAD^+^ for self-renewing. Although NNMT is overexpressed in GSCs, the levels of NAD^+^ are not influenced because NAMPT is upregulated in GSCs. Altogether, NNMT is a factor promoting GBM development, and consistent with this idea, patients of glioblastoma with lower NNMT levels would have longer OS and DFS time (Fig. 5A) [57]. In the future, the combinatorial treatment with inhibitors for NNMT and NAMPT may be a breakthrough for GBM therapy.

### NNMT correlates with Hedgehog pathway in pancreatic adenocarcinoma (PAAD)

Pancreatic adenocarcinomas (PAADs) are extremely aggressive cancers, and the majority of pancreatic cancers are pancreatic ductal adenocarcinomas with a 5-year survival rate of less than 7% [76, 77]. PAAD is accompanied by a desmoplasia which forces PAAD cells to adapt to a severe hypoxic microenvironment, where hypoxia triggers HIF-1-dependent and Sonic Hedgehog (SHh)-mediated tumor–stromal interactions [78]. To survive and proliferate in this condition, PAAD cells trigger a series of specific metabolic pathways to meet their huge energetic and biomass demands, such as enhanced glucose and glutamine metabolism, which are driven by the *KRAS* oncogene [79]. NNMT level in PAAD is much higher than that of normal pancreatic tissue, correlating with unfavorable clinicopathological features, and is proposed as an independent prognosticator of patients’ survival [80]. As Fig. 5A shows, the NNMT level is negatively related to the OS and DFS time of PAAD patients [57]. However, the factors that promote NNMT expression in PAAD are undiscovered. A microarray analysis performed on non-malignant human pancreatic cells overexpressing Gli1 (Hedgehog transcription factor) identified NNMT as one of the 26 potential target genes of the Hedgehog signaling pathway [81]. Considering that the Hedgehog pathway is implicated in the initiation and maintenance of PAAD [82] and NNMT is a potential downstream target gene of Hedgehog, the elevation of NNMT level in PAAD may be a result of the continuously activated Hedgehog signaling pathway. It has been well-demonstrated that upregulation of NNMT enhanced proliferation, migration, and invasion of PANC-1 cells, and vice versa [83]. In aggregate, NNMT performs a pro-tumor role in PAAD as a downstream target of aberrantly activated Hedgehog signaling, but the concrete mechanisms urgently need to be elucidated, which will aid in discovering novel therapeutics and treatments for PAAD patients.

In KIRC, NNMT was proved to promote tumor cell invasion via PI3K/Akt/SP1/MMP-2 pathway, and coincidently, the PI3K/AKT/mTOR signaling pathway is also frequently activated in PAAD [84]. Connecting these facts, NNMT may also trigger a surge of MMP-2 through PI3K/AKT signaling and thus promote the invasion capacity of PAAD cells. In fact, MMP-2 was upregulated in several human pancreatic cancer cell lines and SHh overexpression in PAAD cells activated the Hedgehog signaling pathway in stromal pancreatic stellate cells rather than activating PAAD cells directly to augment the output of MMP-2 as well as MMP-9 [85, 86]. From this point of view, a high NNMT level is also observed in high-grade serous carcinoma and the stroma of breast and colon cancer. Likewise, PAAD also has a dense stroma filled with fibroblasts and immune cells, which contribute to low carcinoma cellularity [61, 87]. The PAAD stroma supports tumor growth and promotes metastasis, serving as a physical barrier to drug delivery [88]. It is putative that NNMT may also be highly expressed in the stroma of PAAD and regulate gene expression patterns to facilitate the formation of a cancer-associated fibroblast phenotype. Stromal expression of NNMT may serve as a vital regulator in the tumor milieu by regulating cytokine secretion, oncogenic matrix formation, and the exchange of growth factors, metabolites as well as exosomes between PAAD cells and stroma cells [61, 89, 90].

### NNMT is downregulated in liver hepatocellular carcinoma (LIHC)

In physiological conditions, hepatocytes express the highest levels of NNMT, indicating a pivotal role of NNMT in the liver. In line with this idea, high expression of NNMT is related to lower triglycerides and serum cholesterol, both in mice and humans [51]. Early work established that 1-MNAM increased Sirt1 protein levels and activity and therefore, suppressed fatty acid and cholesterol synthesis in hepatocytes, as well as decreased liver triglyceride and cholesterol concentrations [51]. LIHC is characterized by a series of metabolic alterations such as increased glucose uptake and lactate production [91, 92], upregulated de novo lipogenesis and NADPH production [93, 94], accelerated glutamine consumption [95], elevated amino acids metabolism, and GSH synthesis [96–99], as well as attenuated TCA cycles and fatty acid β-oxidation [93, 100, 101] when compared with the non-cancerous sample tissues [102]. In consistence with these observations, LIHC cells have a decreased NNMT level and a lower concentration of 1-MNAM, for the purpose of lowering Sirt1 protein. In this case, LIHC cells open the floodgates of fatty acid and cholesterol synthesis, which is advantageous for their growth and malignancy even under hypoxic conditions.

In the previous studies, Kim reported that the expression of NNMT mRNA in hepatocellular carcinoma was significantly lower than that in normal para-carcinoma tissues [103]. The specific mechanisms of NNMT downregulation in LIHC are still unclear. For all we know, the liver is the primary organ of xenobiotic and drug metabolism and clearance in the human body, which expresses plenty of detoxification enzymes as well as transporters related to drug absorption and excretion [104]. Methylation is regarded as a minor detoxification process and thus the activities of liver detoxification enzymes like *N*-methyltransferases will affect the detoxification function of the liver, and then affect the degree of damage to the liver. At present, NNMT and CYP2E1 are the only two enzymes that act as NAM clearance enzymes [7], and both of them are classified into ADME genes which are involved in drug Absorption, Distribution, Metabolism, and Excretion by the PharmaADME Consortium (http://www.pharmaadme.org) [104]. As a result, it is not surprising that they express the highest levels in hepatocytes among all the human tissues. More interestingly, *NNMT* and *CYP2E1* are both listed as the topmost downregulated genes in LIHC [105]. Decreased expression of genes encoding detoxification enzymes (CYPs) is a recognized phenomenon in LIHC and is partially due to the dedifferentiation of cancer cells [106–108]. Therefore, the decrease of the NNMT level may also be a contributing factor that promotes the occurrence and progression of LIHC due to an impairment in the detoxification capability of the liver.

Further, methionine adenosyltransferase, *MAT1A* is expressed predominantly in the adult liver but observably decrease in LIHC patients, implying an elevation of methionine levels and a reduction of SAM levels in LIHC cells [57, 109]. Actually, serum level of methionine is dramatically increased in LIHC patients compared with healthy subjects [110, 111], and mice lacking Mat1a have reduced hepatic SAM levels and developed oxidative stress, steatohepatitis, and hepatocellular carcinoma [112]. Probably, NNMT downregulation is a consequence concomitantly followed by the reduction of SAM synthesis in LIHC. On the whole, NNMT downregulation in LIHC relieves the inhibition of fatty acid and cholesterol synthesis in tumor cells, promotes tumor progression by causing a detoxification capability loss, and reduces the consumption of SAM which makes SAM available for other crucial reactions.

### NNMT is downregulated in pheochromocytoma and paraganglioma (PCPG)

Pheochromocytoma and paragangliomas (PCPGs) are rare and unique tumors originating from the neural crest-derived chromaffin cells in the adrenal medulla or paraganglia [113]. Chromaffin cell tumors within the adrenal glands are termed pheochromocytomas while tumors arising from extra-adrenal chromaffin cells are defined as paragangliomas [114]. The most distinct feature of PCPGs is they often produce and secrete catecholamines including adrenaline, noradrenaline, and dopamine, among which the synthesis of adrenaline is catalyzed by phenylethanolamine *N*-methyltransferase (PNMT) and needs SAM as a co-substrate [115]. Intriguingly, the transcriptional profiling of tumors showed that PCPGs can be divided into two clusters: a “pseudohypoxic” cluster that includes tumors with von Hippel–Lindau tumor suppressor (VHL) and succinate dehydrogenase (SDHx) mutations and an “activated tyrosine kinase” cluster, mainly associated with aberrations in ret proto-oncogene(RET) and neurofibromatosis type-1 (NF1) [114]. PCPGs manifest a strong hypoxic-like signaling, abnormal mitochondrial morphology as well as DNA hypermethylation, which is increasingly recognized in SDHx-mutated PCPG [116]. Besides, early metabolomic studies showed that SDHx-associated tumors possessed high levels of methionine that might be consistent with DNA hypermethylation [117]. It is likely that NNMT under-expression helps PCPG cells to sustain a high SAM level, leading to the maintenance of DNA hypermethylation and catecholamines formation. Moreover, it has been demonstrated that the quantity of NAD^+^ in cluster I PCPGs was found to be 2.7-fold higher than that in cluster II PCPGs and elevated NAD^+^ supports DNA repair pathway, resulting in DNA damage detoxification in SDHB-associated PCPGs [118]. So far, we have no idea about whether NNMT is preferentially overexpressed in cluster I PCPGs, but it can be assumed that PCPGs need NAD^+^ and SAM, which can be achieved by the downregulation of NNMT. High NNMT levels are not good for PCPGs to maintain their properties. As Fig. 5B shows, when NNMT expression is higher, OS of patients with PCPG is longer [57].

## NNMT is a promising tumor biomarker and a therapeutic target

In addition to the tumors mentioned above, it was found that the concentrations of serum NNMT in non-small cell lung cancer (NSCLC) patients were significantly higher than that of healthy people or patients suffering from the chronic obstructive pulmonary disease (COPD) [119]. Additionally, NNMT levels were also observably elevated in the serum of CRC patients [120] and the saliva of OSCC patients [121] compared with healthy people. A few studies indicated that NNMT was more sensitive than carcinoembryonic antigen (CEA) for the detection of NSCLC and colorectal cancer (CRC), and combined testing of NNMT and CEA in serum can improve the diagnosis accuracy [119, 122–124]. A recent study also indicated that the serum NNMT, L-Plastin (LCP1), non-metastatic cells 1 protein (NM23A) triple marker assay could be a powerful diagnostic assay for the early detection of renal cell carcinoma [125]. The feasible reason for NNMT secreted in the serum of NSCLC and CRC patients or the saliva of OSCC patients is that NNMT can be transported out of the cells via exosomes probably, like the way of producing extracellular NAMPT [126]. It has been reported recently that cancer stem cell (CSC) enrichment from multiple cancer cell lines was accompanied by an enhancement of NNMT expression [127]. It is interesting and necessary to explore the mechanisms between NNMT and CSCs. A meta-analysis of 3340 patients with solid tumors from nine published studies indicated that elevated NNMT levels may be a poor prognostic biomarker for patients with solid tumors [128]. A large body of evidence suggested that NNMT is tightly related to cell proliferation [129], migration [130], invasion [36], progression, and differentiation [131] in some tumors. There is an abundance of evidence suggesting that the level of NNMT has important reference values for tumor metastasis [132], prognosis assessment, and survival judgment [133–135]. Herein, it is necessary to carefully investigate whether NNMT can be used as a diagnostic biomarker for some tumors. Meanwhile, it is important and meaningful to develop kits for NNMT detection in cancer diagnosis and to discover NNMT inhibitors for cancer therapy.

## Conclusions and perspectives

**Fig. 6:**
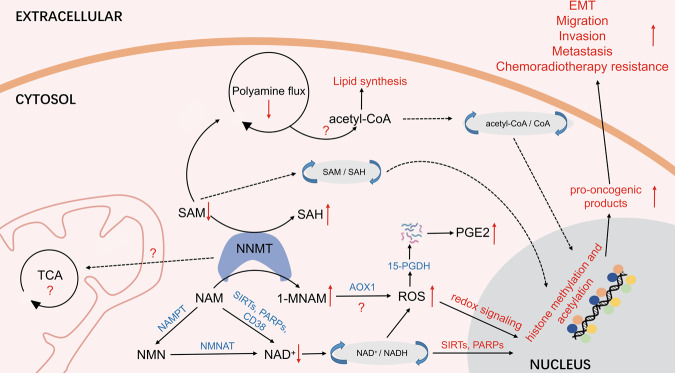
Possible mechanisms of NNMT-promoted malignant progression of some tumors. High expression of NNMT may contribute to the downregulation of SAM, NAM, and NAD^+^ levels. The reduction of SAM may lead to the accumulation of acetyl-CoA for lipid synthesis in some types of tumors. Then the changes of SAM and acetyl-CoA may trigger the epigenetics remodeling and affect gene expression, which brings about the production of pro-tumor products. On the other hand, the decline of NAM and NAD^+^ may increase ROS and influence enzymes activities like SIRTs and PARPs through a switch between NAD^+^ and NADH. Oxidative stress results in the degradation of 15-PGDH and accumulation of PGE2, an important pro-inflammatory and immunosuppressive factor. These changes may eventually promote EMT, migration, invasion, metastasis, and chemoradiotherapy resistance. Inexplicably, the product of NNMT catalyzed reaction, 1-MNAM, is a bioactive molecule but now we know little about it. 1-MANM may have target proteins or pathways and play roles in tumors. Question marks mean that the mechanisms of corresponding pathways are still unknown.

NNMT is either highly expressed or under-expressed in different tumors and plays complex roles in cancer progression by affecting cellular metabolism and epigenetics remodeling. High expression of NNMT promotes proliferation and invasion in some tumors, while low expression of NNMT in some other tumors may be an adjustment measure for maintaining the specific tumor cell phenotype. It is conceivable that metabolic dysregulation promotes cancer progression through not only energy production, but also epigenetic reprogramming. As a metabolic enzyme consuming NAM and SAM, the expression and activity of NNMT can regulate multiple metabolic pathways and promote epigenetic remodeling in some tumors by affecting the levels of key metabolites such as NAD^+^ and SAM, even acetyl-CoA probably (Fig. 6). But more subtle mechanisms of NNMT in tumors remain elusive and additional work that demonstrates the molecular and cellular mechanisms whereby NNMT plays dual roles in different tumor cells is urgently awaited. Detailed characterization of pro-tumor or antitumor effects of NNMT may pave the way for the development of inhibitors and treatment in tumor patients with high or low NNMT expression. In combination, our understanding of NNMT has grown remarkably in the last two decades and the pace of relevant research is accelerating. Shortly, more discoveries about NNMT certainly will come.

## Data Availability

All data generated or analyzed during this study are included in this published article.
